# The Investigations of Nitric Oxide Influence on Lifespan of Fruit Fly *D. melanogaster* Transgenic Strain dNOS4

**DOI:** 10.5195/cajgh.2014.153

**Published:** 2014-12-12

**Authors:** Mamura Begmanova, Nata Mit, Anara Amirgaliyeva, A. Tolebayeva, Leyla Djansugurova

**Affiliations:** Institute of General Genetics and Cytology, Almaty, Kazakhstan

**Keywords:** aging, longevity, nitric oxide synthase transgene, lifespan

## Abstract

**Introduction:**

Aging and longevity control are among the greatest problems in biology and medicine. The fruit fly *Drosophila melanogaster* is a nice model organism for longevity investigations because of its biological features. Many *D. melanogaster* genes have their orthologs, similar in other eukaryotes, including human. The role of nitric oxide (NO) in the *D. melanogaster* lifespan has been analyzed.

**Methods:**

Virgin flies of dNOS4 transgenic strain were used for the experiment. This strain contains non-functional additional copies of nitric oxide synthase (NOS) gene under heat shock promoter. For promoter activation, transgenic flies on their second day of life were exposed to heat shock (37°C) for an hour. After heat shock, flies were maintained on standard medium temperatures at 25°C, with females separate from males. Two types of control were used: Oregon R wild-type strain and Oregon R strain exposed to heat shock. The average lifespan was evaluated.

**Results:**

It was revealed that the longevity of females was significantly higher than males in each series of experiments (*p* < 0.05). The survival rate of females and males was similar in the first month of their life, but in the second month the mortality among males was much higher than among females in all series of experiments. The average lifespan of dNOS4 imago was 31 days (34 days for females and 28 days for males), maximum lifespan was 63 days. In controls, the average lifespan of Oregon R flies was 54 days (58 days for females and 50 days for males), and the maximum lifespan was 94 days. The average lifespan of Oregon R flies exposed to heat shock was 45 days (48 days for females and 41 days for males), and the maximum lifespan was 72 days. The difference between average lifespan in all studied groups is statistically significant (*p* < 0.05).

**Conclusion:**

Thus, NOS-transgene activation results in formation of non-functional dNOS4-transcripts and NO deficiency. In turn, NO deficiency decreases dNOS4 imago lifespan.

